# Notes and News

**Published:** 1900-04-14

**Authors:** 


### Notes and News.

### HOSPITAL MEETINGS.

**French Hospital.—Annual Meeting on Thursday, April 12th.**

The Congress on Tuberculosis opens at Naples on April 18th.

Dr. W. S. Church has been re-elected President of the Royal College of Physicians.

By the express wish of the Queen, the City of Dublin Hospital will henceforward bear the prefix of "Royal" to its title.

Sir James Reid, physician to the Queen, has been made Honorary Fellow of the Royal College of Physicians of Ireland.

Since the opening of the London School of Tropical Medicine in October, 1898, 52 students have passed through the course of study.

A large amount of typhoid prevails amongst the Boer prisoners, and it is stated that the disease is spreading amongst the Cape Town population.

Mrs. B. Moore, M.A., Professor of Physiology at Sheffield Medical School, late Assistant Professor of Physiology, University College, London, has been appointed Lecturer on Physiology at the Charing Cross Hospital Medical School.

As the result of the recent conference of the National Association for Promoting the Welfare of the Feeble-minded, the association undertakes to promote the establishment of institutions for the care of the feeble-minded throughout the country. The homes are to be industrial, and assisted by voluntary contributions.

A new school of medicine has been established at Tauris, in Persia, by a Court physician, a graduate of the Paris Faculty. Three professors, each having an assistant, compose the teaching staff. Only young Mussulmans will be accepted as students. The education will be general as well as medical.

There have been a considerable number of cases of plague in Sydney. Upwards of ninety have been reported. The death-rate has been considerable.

The sick and wounded are now arriving in large numbers, and extra military hospital accommodation is being prepared. At Aldershot huts for 700 patients are preparing. They will be of corrugated iron with match-board lining. The Herbert Hospital is also being supplemented in the same manner, and at Netley there are the German Red Cross huts.

We have remarked before in these columns that those responsible for the conduct of institutions are mostly oblivious of the goodwill shown towards them by newspapers through notices which appear of matters affecting their interests. It is therefore especially pleasing to editors when appreciation is expressed in the graceful manner which the Italian Hospital has adopted towards those journals which recently gave an account of the opening of its new buildings.

Liverpool seems to be following the example of St. Helens in trying to lessen in some degree a large infant mortality by instituting depôts for the sale of sterilised milk. The Liverpool Corporation have voted £1,000 to be placed at the disposal of the Public Health Department to establish the system. Bottles will be provided at the various depôts, which will have to be returned for cleansing and refilling, and it is hoped that the mothers will gradually become educated in the proper feeding of their children.

The eulogies bestowed upon the Army Medical department of the forces in Africa in the House of Commons during the debate on supplies, cannot fail to gratify those who may not have realised how much their self-sacrificing, heroic, and efficient conduct has been appreciated at home. Mr. Wyndham stated that the House could not pay too high a tribute to the fortitude, courage, and efficiency of the medical officers in the field, and Dr. Farquharson declared that the Royal Army Medical Corps had not been the subject of a single complaint since the war began.

A well-known man of science has just passed away in the person of Dr. St. George Mivart. He has been especially prominent recently through his correspondence with Cardinal Vaughan, arising out of his endeavour to reconcile scientific principles and Catholicism. Dr. Mivart died at the age of 72. He commenced his education under the late Professor Pritchard, at Clapham, and passed thence to Harrow, King's College, and St. Mary's Roman Catholic College at Oscott. He was called to the Bar, but left it to pursue scientific research. He became Lecturer at St. Mary's Hospital, Fellow of the Royal Society, Secretary and Vice-President of the Linnæan Society, and Professor of Biology at University College. He was created a Ph.D. Rome and M.D. of the University of Louvain, and Professor of the Philosophy of Natural History at the same University. He contributed a considerable amount to scientific literature, and strongly combated some of Darwin's conclusions.

